# Virtual consultations: the experience of oncology and palliative care healthcare professionals

**DOI:** 10.1186/s12904-024-01400-y

**Published:** 2024-05-02

**Authors:** Heledd Lewis, Mark Taubert, Annmarie Nelson

**Affiliations:** 1 Velindre University NHS Trust, Cardiff, CF14 2TL UK; 2 School of Medicine, Marie Curie Palliative Care Research Centre, Cardiff, UK

**Keywords:** End of life care, Symptoms and symptom management, Supportive care, Covid-19, Communication, Clinical assessment, Virtual

## Abstract

**Objectives:**

To maintain continuity of care during the Covid-19 pandemic, virtual consultations (VC) became the mainstay of patient-healthcare practitioner interactions. The aim of this study was to explore the views of oncology and palliative care healthcare professionals (HCPs) regarding the medium of VC.

**Method:**

A cross sectional mixed methodology observational study of oncology and palliative care HCPs, analysed via an inductive thematic approach. This was undertaken in accordance with relevant guidelines and regulations.

**Results:**

87 surveys were completed. Three master themes were identified. Personal, professional, and familial factors including patient age, illness and VC skillset all influenced practitioner’s experience of VC. Relationships and connection were highlighted by survey respondents as important influences, with a perception that VC could reduce usual relationships with patients, compared to previous face-to-face consults. There was a perceived loss in these domains with VC. Sharing bad news and having challenging conversations was seen as particularly difficult via VC. Many survey respondents emphasized that they preferred to have first time consultations face-to-face, and not virtually. Within the domain of logistical and practical implications reduced travel and increased accessibility were seen as a significant benefit of VC. The inability to examine patients and concerns regarding missing clinical signs was emphasised as a significant worry, alongside the challenges faced with occasionally failing technology.

**Conclusion:**

VC were felt to have a role for those patients who are already known to professionals, where there was an established relationship. VC for difficult discussions and for unstable patients were felt to be inadequate. Triaging patient suitability prior to offering VC, with emphasis on the importance of patient choice, was seen as a priority in this new era of VC.

**Supplementary Information:**

The online version contains supplementary material available at 10.1186/s12904-024-01400-y.

## Introduction

During the Covid-19 pandemic, healthcare professionals (HCP) and patients have had to familiarise themselves with virtual consultations (VC) and its accompanying technology, to ensure continuity of care. This was especially important given governmental restriction on free movement/interactions and shielding guidance [1]. There is a growing body of evidence published in the last year, driven by the pandemic, that has encouraged research using VCs in the field of palliative care and oncology. Both specialities can be emotionally demanding for healthcare professionals, with HCPs often managing severely unwell patients. The work involves complex decision making, managing legal and ethical issues, in addition to caring for patients at the end of life [2]. The sudden need for HCPs to translate their usual delivery of care onto a virtual platform, and their experience on the challenges and merits inherent in this change has been explored in this research project. The role that VC modalities may have in the future will be discussed, with the aim of considering if and how VCs can supplement the care provided to patients within these fields in the future.

## Methods

### Aims

To explore the experience of virtual consultations from the healthcare professional’s perspective in the delivery of oncological and palliative care.

### Rationale for methodology

The study was a cross-sectional mixed methodology observational study using a survey as the data collection tool. To achieve this, an interpretative paradigm was used to understand and discover patterns in the data, [3] which were then analysed via an inductive thematic approach. A mixed methodological approach was chosen consisting of two components within the study: a quantitative method of data collection and qualitative section of thematic analysis. The quantitative data were analysed using descriptive statistics, mainly encompassing the demographic data and participant information relating to the sample.

### Participants

Purposive sampling was used within the study as the sample needed to have a role in a specific field of practice and experience in order to answer the research question [4]. The aim of the study was to look solely at the experience of healthcare professionals within the field of oncology and palliative care regarding virtual consultations. 48.8% of respondents were oncology HCPs and 51.2% were palliative medicine HCPs. 63.2% were working in the hospital setting (of these 85.5% were based in a cancer centre setting), 8% in the hospice setting and 27.6% in the community setting.

See Appendix 1 and 3: Inclusion and exclusion criteria and table of results.

### Recruitment

The survey was distributed online using Jisc online surveys® between the 1st of March 2022 and the 1st of April 2022. The link to the online survey was disseminated via the researcher’s personal social media pages on the 1st March 2022, including Facebook® and Twitter®. Furthermore, posters in the local hospitals and hospice settings within healthcare professional areas were used to inform about the survey, and staff were encouraged to invite other colleagues.

The survey link was mailed, shared and reposted via online media by professional contacts of the researcher. The post included the link and a banner advertisement. Palliative care and oncology forums, as well as a conference and local grand round were also used to maximise healthcare professional engagement.

Data were analysed as surveys came in, and broad themes (see Results) were identified. Themes were categorised and sub-categorised. At the point of reaching 76 surveys, no new themes were emerging, and therefore a decision was made to close the survey at the pre-determined time, without the need for a further extension. All remaining surveys that came in until the closing date were included, and again, no new themes emerged.

### Enhancing response rate

The survey link was also shared via a QR code at a palliative care congress in March 2022 to gain further respondents. Response rate was also enhanced by “re-tweeting” or re-posting the link during the period the survey was open, with the intention of this being further reposted and creating a snowball effect.

### Survey development and data collection

A cross sectional descriptive survey using Jisc online surveys® was developed, with a 20-minute completion time and consisting of three sections. The survey was developed for this study and not published elsewhere. The survey was developed by the first author and refined and edited (see Appendix 2). It was pilot-tested on 3 healthcare professionals and then further augmented, based on this pilot. The first section included the demographics and participant background information. Section two included a variety of question modalities, mainly focusing on the barriers and benefits in the participants’ experience of virtual consultations, and section three further focused on the breaking bad news aspect, relationship factors and future of virtual consultations going forward, with further emphasis on free text responses. The survey included a mixture of multiple-choice questions, dichotomous questions, open-ended questions with free text, Likert scales and rank order questions.

### Reflexivity

Reflexivity is vital within qualitative research and involves the “process of reflecting critically on oneself as a researcher.” [5] A documented anonymised log throughout the study period was kept demonstrating progression and development. As the survey was published on the researchers own social media accounts, it is acknowledged that the viewers and potential participants to the research project would know the researcher, or the researcher’s contacts shared a common set of interests. The benefit of this in obtaining a purposive sample is clear, but also highlights the importance of considering the role of the researcher within the research process [6]. 

### Data analysis

Following the closure of the survey, the data were exported to SPSS statistics for analysis.

Simple descriptive statistics were used to analyse the quantitative elements of the survey, including the demographic data, closed ended questions, Likert scales, and multiple-choice questions. Percentages were calculated to one decimal point and limited data cleaning occurred to ensure consistency in the documentation of the data. Subgroup analysis was undertaken when relevant to the question in hand, but no subgroup comparison occurred between oncologists and palliative medicine healthcare professionals or between professional roles within this research study, although this could be considered in future work.

The written text and comments within the open-ended free text responses were evaluated via an inductive approach, using a reflexive thematic analysis as described by Braun and Clark and themes were derived from the data [7]. 

The qualitative aspect of data analysis was undertaken manually. The codes were distributed into a coding tree of themes and subthemes, then reviewed and refined at regular intervals. Further validity was gained by reviewing the final themes and subthemes with a supervisor [8]. 

All surveys were analysed, regardless of their full completion. Non-response to questions was minimal and considered within the data analysis.

## Results

87 surveys were submitted in total between the 1st March 2022-1st April 2022. No survey data were excluded.

See appendix 3: Table for demographic and quantitative data.

Analysis of free text data.

**Fig. 1:**
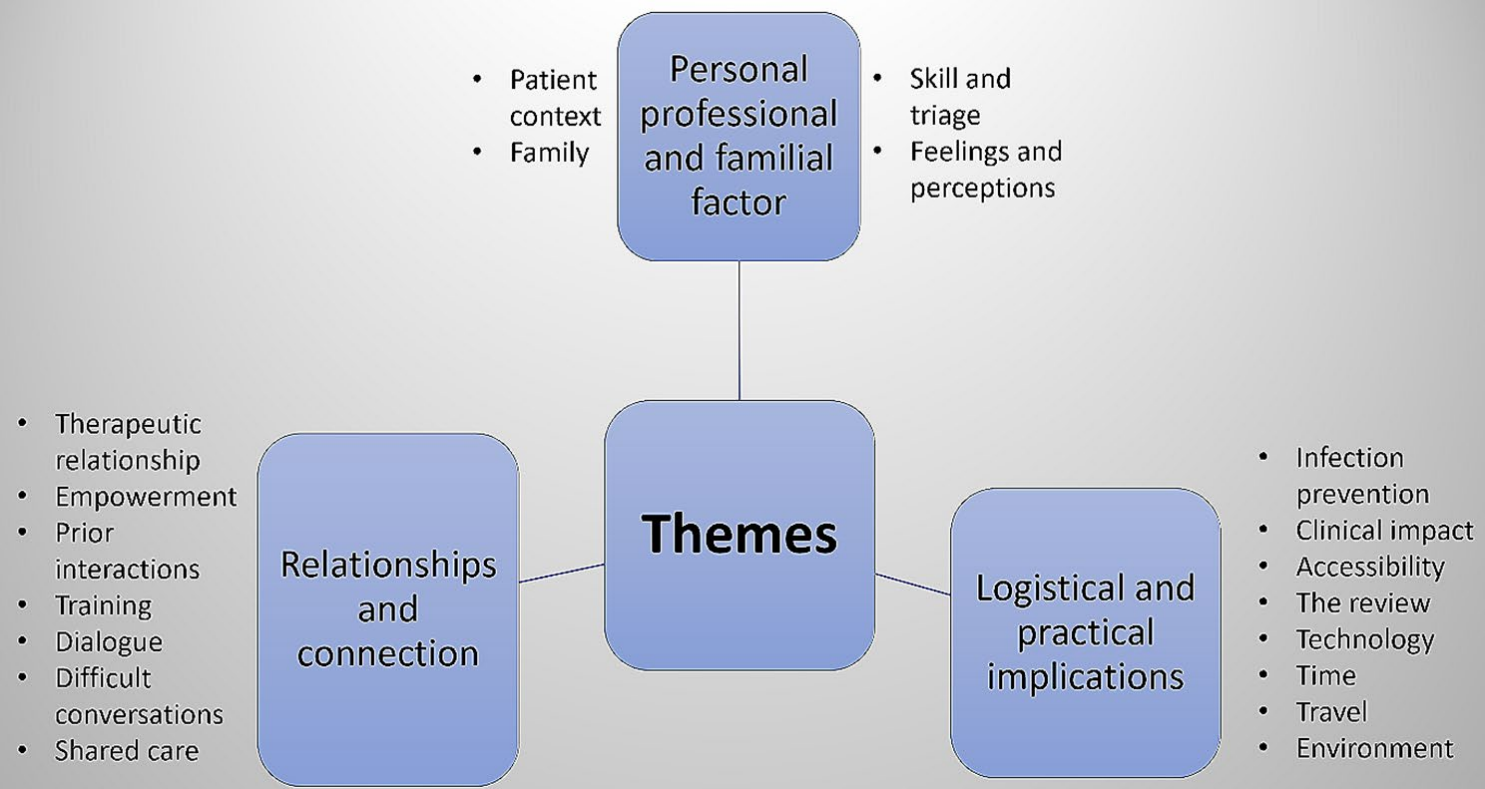
Overview of the themes

Figure 1 below gives an overview of the themes formed following an inductive thematic analysis of the free text comments on the experience of oncology and palliative care health care professionals of using VC modalities.

### Theme 1: Personal, professional, and familial factors

#### Subtheme: Patient context

An awareness of the patient’s context was an important consideration when thinking of the benefits of VC. This related to the context of an unwell patient, who required review, but was too unwell to physically attend a clinical setting. Some patients were too frail to attend in person but benefited from the ability to be reviewed via VC. 53 respondents (61.6%) considered VC’s beneficial when patients had difficulty leaving their home, or felt too unwell or tired to travel.

On the other hand, other respondents felt that determining the patients context, or how unwell they were via VC as a modality of communication was quite challenging. They felt the needs of an unwell patient to be quite different to a less unwell patient.

*“In my experience it is very difficult to assess unwell patients and give psychological support virtually” (P1 Oncology SpR)*.

The ability for patients who were working to be reviewed within their working day, without the need to take leave from work was another clear benefit of VC, and there was an assertion that VC was very suitable for younger patients, compared with older patients.

*“Also many patients can do this in between work (for ones who are working); whereas for F2F, they have to take generally the whole day off” (P64 Oncology Consultant)*.

*“Challenges are often patient acceptability- I find the younger age group are more familiar with its use, older group generally not so comfortable” (P68 PC Consultant)*.

#### Subtheme: Skill and triage

49 (56.8%) of the participants felt confident consulting virtually but felt that patient uncertainty and skill could be a barrier. One participant felt that their own skill and confidence improved with growing exposure and experience in the VC modality. Similarly, another respondent felt that the patient’s confidence appeared to improve with use.

*“Patients frequently lack the confidence to be able to use technology to a sufficient level”(P48 PC CNS)*.

*“Competence increases with more use, and the ‘virtual’ barriers reduce considerably” (PC Social worker)*.

58 (55.8%) of respondents found that the patients’ lack of confidence with technology was somewhat or very challenging when considering barriers to VC.

When considering the skill of breaking bad news, one respondent felt that the skills required when breaking bad news in a VC setting was different to those required when reviewing a patient face to face, highlighting the possible need for increased training and awareness of what is necessary from the professionals perspective.

#### Subtheme: Feelings and perception

From the healthcare professional perspective, when considering the benefits of VC, there was a significant emphasis on the benefit of VC on wellbeing and mental health during the uncertain time of the pandemic, as some healthcare workers were shielding because of their own health or vulnerable family members. Participants felt that the patients were more comfortable using VC, being in their own space, own homes, and surroundings and that this in fact put them at ease. Reduced apprehension was felt to positively impact the patient-practitioner relationship.

*“As someone who shielded with a vulnerable child, being able to continue with seeing my patients made a very real difference to my overall mental well-being at a time of great uncertainty.”(P15 PC Consultant)*.

*“Improves and deepens (the healthcare professional-patient relationship). A sense of a very safe private space to develop a relationship, whilst feeling safe and relaxed in their own home.” (P32 PC Consultant)*.

One participant was concerned that patients and relatives may feel offended, angry and insulted by the offer of VC, especially when patients were dying.

*“Patients/family members find it offensive/insensitive and an indication that HCP can’t be bothered to see the dying person” (P68 PC Consultant)*.

#### Subtheme: Family

When considering the benefits of VC, the majority, 48 (55.8%) of participants did express that the improved ability to ‘meet’ with family members and significant others via VC, to be a slightly or very important benefit to consider. This was considered from the point of view of the visiting restricitions that were in place due to Covid-19, limiting or forbidding visitors to enter the hospital setting, and from the point of view of enabling families from large geographical areas to be part of discussions and reviews.

*“Multiple family members in multiple locations being able to join in”(P28 PC CNS)*.

Although the ability to reach families from a wider geographical area for their involvement in consultations was a benefit, others felt that it did bring communication challenges with it.

*“Its great that multiple family members can attend and participate, but sometimes they sit out of view and contribute and you can’t see them, or see their body language, and therefore it can be more challenging to meaningfully interact with them, like you can when everyone is in the same room”(P45 PC SpR)*.

Participants also recognised that regular physical hospital appointments for those needing oncological or palliative care can put a momentous strain on family members and carers, and that virtual was perhaps an easier option for ‘joining in’ on a consult.

*“Reduces burden on carer having to bring patient to clinic”(P40 PC CNS)*.

### Theme 2: Relationships and connection

#### Subtheme: Empowerment

Participants regularly mentioned the importance of patient choice, i.e. patients as decision makers who choose the mode of consultation they would like to have. The choice of modality could therefore enable them to undertake a consultation that they were most comfortable with.

*“They usually appreciate the variety in modes of consultation. This fosters better relationships. Patients need to be assured there are also equal decision makers in having choice.”(P64 Oncology Consultant)*.

Another participant commented on the power imbalance that can be seen in healthcare, between the professional and patient within the physical space, and by the nature of coming to a clinical environment, how one may feel intimidated. This correlates with previous comments regarding a sense that patients feel more at ease and less anxious using VC modalities.

*“(Virtual consultation) can support a more egalitarian relationship, patient not coming into ‘my space’ and can reduce potential power imbalance.” (P12 Oncology Psychologist)*.

#### Subtheme: Therapeutic relationship

The therapeutic relationship between the professional and patient was discussed throughout the data by participants and the ways in which VC can impact on this relationship. The characteristics of a therapeutic relationship referred to in the text involved trust, confidence, empathy, bond, rapport, respect, and touch. Aspects referred to the challenges faced by HCP when needing to console patients, and the inability to do this adequately via VC. They expressed missing this element of patient contact especially when breaking bad news or giving difficult information.

*“Main challenge has been difficulty reassuring the patient if becoming upset, especially if video breaking up” (P17 Oncology SALT)*.

*”I perceive a more meaningful relationship with patients following face to face assessment” (P48 PC CNS)*.

The concept of rapport was reiterated on several occasions by many of the participants, and how consulting virtually impacted on the ability establish and build on rapport and trust.

*“I think they reduced the ease of building rapport, but they are far better than a mere telephone call, because you get to put a face to a name” (P16 Oncology OT)*.

Similarly, participants also described a possible feeling of reluctance from patients to ask key questions during a VC, which clearly is a concern when considering the understanding of treatment decisions. This was often exacerbated by technological problems, such as poor signal.

*“There can be a level of intensity of virtual which may not allow important questions to be asked” (P3 Oncology CNS)*.

#### Subtheme: Shared Care

The concept of shared care applies to several aspects of the consultation. It can relate to shared care between the MDT, with other sectors of care e.g. the acute sector or primary care, and shared care with wider teams of professionals, and specialities.

With VC, links can be sent to other team members or colleagues caring for the patient to attend meetings and discussions to ensure input from all specialties and disciplines involved.

36 (41.3%) of respondents felt the ability to review patients with multiple members of an MDT, to be a slightly or very important benefit of VC.

With restrictions in place due to infection control measures during the pandemic, MDT meetings could be held virtually, which participants felt was another benefit of VC.

*“Ability to continue MDT clinics despite social distancing requirements, ensured patients received the care they needed from full MDT” (P86 Oncology Speech and Language Therapist SALT)*.

Participants expressed situations where this had worked well with young/transitional patients, where MDT input was required and in initial assessments with Clinical Nurse Specialists (CNSs) and therapist for assessment and review. Similarly, other professionals and specialists were able to join and aid discussions, by ‘dialling in’ and not physically having to join in person, which improved.

*“Can review inpatients in other hospitals much more efficiently, so can support acute care” (P47 Oncology Consultant)*.

#### Subtheme: Difficult conversations

**Fig. 2:**
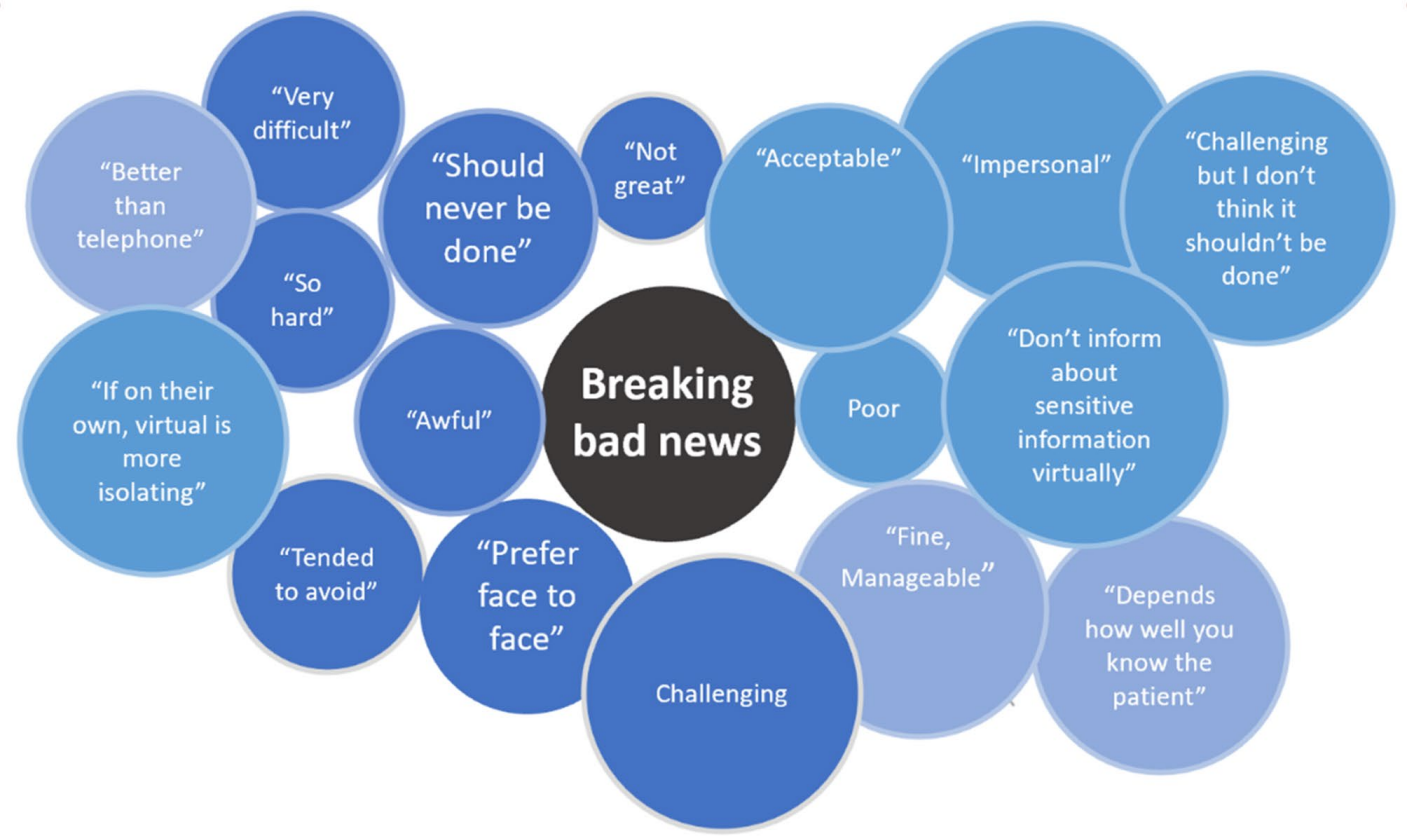
Quotes from healthcare professionals experience of breaking bad news discussions with patients during VC

When considering participant experience of breaking bad news in a VC, 37(64.9%) felt negative towards the process, 13 (22.8%) had mixed feelings, and 7(12.2%) felt positive about their experiences. Figure 2 summarises a number of the key phrases as words used to describe the process of breaking bad news using VC.

When participants were asked which method of consulting works best for them, when breaking bad news, 79 (100%) of the 79 respondents expressed that they would nearly always prefer face to face consultations.

Participants felt that using a VC modality to break bad news was better than on the phone but would generally prefer to undertake this task face to face, indicating a preference hierarchy.

Some expressed that bad news consultations were all undertaken face to face in their place of work, and VC modalities were never used for this purpose, indirectly highlighting the inappropriateness felt by some HCPs of using VC to have such discussions. Participants also emphasised the importance of triage to determine which patients would accept difficult conversations via VC.

*“The key in my mind is to choose mode of consultations personalised to the patient in discussion, and not have a blanket rule to follow blindly” (P64 Oncology Consultant)*.

Several participants expressed that breaking bad news using VC is more appropriate with patients already known to the HCP i.e. where a prior relationship had already been formed.

*“For patients I already know well and have a good rapport with, it has been acceptable, I think. I would be very hesitant in embarking on this if I did not know the patient or the situation well.” (P39 Oncology Consultant)*.

Participants expressed the challenges with communication when breaking bad news virtually and aspects of commuication skills that are lost in this situation.

*“Harder to pick up on cues from them, as to pace of information gathering. If they are on their own, virtual is more isolating if breaking bad news. Face to face one can allow for silence more readily”(P9 PC Consultant)*.

Technological and internet connection issues also played a role in exacerbating the challenges, causing delays, screens freezing and with responses to questions postponed due to auditory problems or feedback.

“*I had one very challenging consultation where the reception was so bad the patient ended up walking off in frustration, very hard to rectify” (P83 Oncology SALT)*.

#### Subtheme: Dialogue

Given that VC is a mode of consultation and communication, it is unsurprising that a significant proportion of respondents commented on their experiences on that specific theme, highlighting the benefits and challenges inherent within VC communicating.

Participants acknowledged the importance and the difficulties with not being able to pick up nonverbal cues and sense body language when consulting virtually and its impact on effective communication, listening and interpreting. This may be due to technological issues and camera placement, the quality of the camera, distance from the patient and technological delays. On the other hand, some participants also felt that VC was better than telephone consultations, as patients were able to lip read and read their nonverbal cues. Others highlighted the importance of considering visual or hearing barriers that the patient may have which might reduce quality of virtual communications.

*“Communication barrier not being able to see full body language and not being able to comfort in person.”(P33 Oncology SALT)*.

#### Subtheme: Training

29 (33.3%) had training prior to conducting VCs, 58 of the 87 respondents (66.7%) did not. Only 18 participants (20.6%) felt that lack of training in VC had caused challenges when consulting. The training varied from colleague and self-directed training, to formal video tutorials and training via the trusts. The length of training varied from ten minutes to one hour.

25 (92.5%) out of the 27 felt that the training was of benefit to them. Participants described the training to be helpful or somewhat useful. Having training enabled them to support, encourage and train others, including how to use the VC platform.

*“Yes, helped build confidence with kit and applications, as well as giving tips for making it work for patients” (P57 PC Consultant)*.

Participants reflected on the challenges in inviting trainees into virtual clinics.

*“I haven’t probably invited junior doctors/trainees to join me in clinic as much as I did previously, as harder to include them on the screen and also means I’d need to wear a mask which I feel would make the video consultation harder for patient.” (P29 PC Consultant)*.

The above observation was made at a time during the Covid-19 pandemic when mask wearing was essential in shared indoor spaces.

#### Subtheme: Prior interaction

This subtheme relates to the perception that VC was often a less troubled option in patients with whom the HCP had held a prior face-to-face consultation with. Participants expressed that VCs were harder with new patients they had never met face-to-face. This was very much a recurring theme in the data.

*“I think it takes more time to establish a connection and there is definitely something missing if you’ve never met the person****in person***. *Consultations have been much more effective on patients that I have met at least once in clinic” (P31 PC Consultant)*.

Similarly, this impact was seen within communication and breaking bad news situations, participants expressing a reluctance to risk having virtual conversations, without a prior real-world relationship with the patient.

*“Using VC for this is more appropriate with patients already known to me” (P2 PC Consultant)*.

Several participants alluded to the benefit of seeing the patients using several modes of consultation, but the initial consultation would be better conducted ‘in person’, to establish the initial relationship and build rapport, followed by VC consultations for further follow-up.

*“Much harder to establish relationship in the new patient consultation. However, adds flexibility when used appropriately interspersed with face-to-face consultations.” (P59 Oncology SpR)*.

### Theme 3: Logistical and practical implications

#### Subtheme: The review

Table in appendix 4 summarises the assessments undertaken via VC by the various HCP roles.

When asked about the role of VC during the delivery of varying stages of patient management, a significant proportion of the participants felt that VC had a role in patients that required routine follow up, if the patient was well and stable, and if they had already met the patient and had an established relationship with them.

Participants felt that VC was appropriate pre-systemic anti-cancer treatment (SACT) review and whilst undergoing SACT, in addition to radiotherapy review consultations to assess side effects.

On the other hand, some participants felt that *“any difficult discussions should by default be in person in my opinion” (P60 Oncology SpR) and “diagnosis and change in treatment plans i.e. from treatments to best supportive care should be face to face were at all possible” P66 (Oncology CNS)*.

#### Subtheme: Accessibility

Participants commented that the use of VC increases patients’ ability to access palliative care services and advice. Challenges with geographical and physical distance can be overcome in this way, and patients that live a distance from acute hospitals can benefit from specialist input regardless of where they live. In addition to this, as demonstrated above, the use of VC allows increased access to family members/significant others/carers to join in with consultations and discussions, even when they are at work or elsewhere.

*“They will be a fundamental part of service delivery. They widen access to our services. I think they have a role in all stages of the patient journey, however they can be inappropriate for individuals at all stages too. Decisions regarding their use must be individualised” (P45 PC SpR)*.

#### Subtheme: Infection Prevention

In terms of the logistical impact of infection control, as described in the literature review, the role the coronavirus pandemic has played in the requirement and implementation of the use of VC in healthcare as a mode to review patients can not be underestimated in allowing shielding patients to be reviewed at a time of significant anxiety to themselves and their families.

*“It has been beneficial to reduce footfall in the cancer centre and to protect patients during the peaks of the pandemic.” (P16 Oncology OT)*.

#### Subtheme: Clinical impact

Some participants reported difficulty in arranging prescriptions for patients using VC. *N* = 23, (27.3%) had experienced challenges with arranging prescriptions, and of these respondents (*n* = 74) were doctors, specialist nurses and pharmacists- therefore likely prescribers. One participant described the inability *“to issue prescriptions there and then is very frustrating and time consuming”* (P67 Palliative care registrar) and the need to improve accessibility to electronic prescription services.

Several participants described their experience and concerns in missing clinical signs using VC modalities. Some participants felt that they were unable to grasp how well the patient was on a screen, impacting on the holistic clinical picture and decision making. *N* = 56 (65%) of respondents found the inability to examine patients via VC quite challening or very challenging, and of the cohort of doctors surveyed (*n* = 51), *n* = 37 (72.5%) found the inability to examine patients challenging to some degree. Lack of examination also exacerbated patient anxiety and increased need for supportive calls and reassurance.

*“Virtual appointments result in missing important clinical signs or changes in patient condition. Need to see these patients face to face to get a good sense of how they are doing.” (P5 Oncology consultant)*.

#### Subtheme: Environment

Some participants felt that the impact of their working environment negatively affected their experience of VC. This was related to challenges with privacy, lack of physical dedicated space to undertake a VC and having multiple staff members in one room whilst trying to consult with a patient. Others described challenges with background noise in an open plan office space and interruptions.

*“Consultant hospice colleagues lurking off camera in consultation and then popping out halfway through.” (P27 PC Consultant)*.

An occupational therapist discussed the benefits of VC in having the ability to see the patients home and what was needed without having to physically assess, and that this enabled quicker processing of equipment.

*“Able to assess home environment to select appropriate equipment, reduced time to wait for equipment.” (P4 PC OT)*.

#### Subtheme: Time

45 of the participants (52.3%) felt that the ability to review patients quicker and with less notice was a slightly or very important factor to consider when undertaking VC. Partcipants felt that consultations and meetings could be arranged with shorter notice, including less need to book hospital transport or send out letters, which increased flexibility for the patient.

*“It is more efficient in contacting people sooner and saves travelling times and costs” (P44 PC CNS)*.

*“Phone at a time more convenient to patient for them” (P55 PC Social worker)*.

Some participants felt that clinics were more efficient and quicker, with less delays where others felt that VC was more time consuming than telephone consultations due to the need to set up IT and delays related to technology.

*“Sessions with patients were shorter and more time efficient therefore long clinics were completed quicker”(P26 Oncology SALT)*.

#### Subtheme: Technology

Participants were asked about their experience relating to the availability of appropriate access to equipment required to conduct VCs. *N* = 52 (59.8%) expressed that they did have access to appropriate equipment. Others felt that lack or insufficient equipment was a barrier in their experience of consulting virtually. This was related to desktop space, computer/laptop unavailability, headsets (with headphone and microphone), and several MDT members congregating in one space attempting to access a single desktop.

In addition to this *n* = 39 (45.3%) of respondents experienced technical issues with the consulting programme and *n* = 39 (44.8%) had issues with poor internet connection.

*“However, if experiencing a bad internet connection, it can feel frustrating and quite a remote relationship” (P21 PC Consultant)*.

Participants explained that rural areas had difficulty with poor internet connectivity and felt that further work needed to be undertaken to improve internet connections prior to undertaking or wholly relying on VC.

#### Subtheme: Travel

One of the significant benefits participants discussed in their responses was the benefit of VC in reducing the need for travel. This was applicable to the HCP’s themselves and their patients/proxy. Over half, *n* = 48 (55.1%) of respondents considered the reduced travel time for patients and HCP to be a slightly or very important benefit of VC.

For HCPs working in the community, VC meant that staff spent less time travelling around, and one oncology consultant felt that because patients were not travelling, they were able to accommodate more patients in one clinic. It was felt that if the message from the consultation was clear and uncomplicated, travelling long distances can be avoided, in addition to avoiding the burden of parking and waiting.

*“Virtual consultations can be useful in order to avoid travelling especially when the message is straightforward, such as a favourable follow-up scan” (P53 Oncology Consultant)*.

## Discussion

Most participants felt strongly that VC had a role in the future management of patients in oncology and palliative care. 53.5% felt that VCs could replace face to face consultations in approximately half of consultations. Overwhelmingly, 71.2% of participants agreed or strongly agreed that a mixture of face-to-face consultations with VC is the way forward.

Murphy et al. suggested further work was required to gauge what type of ‘typical’ consultation was best suited for the virtual modality, compared to others [8]. This survey was clear in demonstrating that from the HCPs point of view, there was a strong preference for consultations that included follow-up assessments and treatment plans to be offered virtually, as opposed to predominantly face to face within oncology and palliative care. Initial assessments, discussions around change in condition and treatment, and advance care planning conversations were felt to be more appropriate face to face, and difficult conversations were perceived as inappropriate and extremely challenging using VC. There would be significant benefit in reviewing patient experience, and whether feelings were unanimous, as this could have significant implications on how patient follow up is delivered.

Participants also expressed the role VC may have in benefiting HCPs in future, e.g. during times of staff shortages, potential ongoing issues with infection control, and reduced necessity to travel including to and from various clinical sites. It was felt that the environmental impact in reducing the impact of travelling was significant and a big factor for HCPs. Also, eliminating waiting times for patients in busy hospital clinic areas, was seen as a benefit, as well as reducing anxiety by being in the safety of one’s own home, during a virtual consult.

Our research also highlighted discrepancies. For instance, in the subtheme “patient context,” the quantitative data show that most respondents found VC beneficial when patients are unwell, presumably because a car journey to a hospital may be very burdensome for a patient and added to this the wait in a busy and noisy outpatient waiting room. However, the qualitative respondent data presented also highlights that assessing how unwell a person is, can be very difficult when using VC. This contrast may just provide a magnifying glass on some of the wider pros and cons of this newer form of consultation. Respondents did not mention or talk about the potential opportunity of recording conversations, and this is not routine practice from the HCPs point of view, but may be something that patients are already doing, whether openly or covertly.

Some felt that there needed to be a greater awareness of technology inequality that exists, therefore VC may not be appropriate for all. The pandemic highlighted the issues around a “digital divide” and the inequality around access to technology, which includes equipment and internet connectivity [8]. This as an opportunity for policy makers to acknowledge and address changes to the inequity in access to technology. They also suggested telehealth hubs, potentially in rural areas, therefore centralising technology more to ensure access to all.

There was a strong belief that the future of VC lies within a hybrid approach of using a mixture of modalities, as was seen in the systematic review by Murphy et al. [9]. Similarly, those that were interested in continuing with telehealth reported the blended approach (face-to-face consultations in addition to virtual) as the best solution [8]. The importance of individual choice and patient empowerment came out as a key factor for HCPs within our survey in allowing the patient themselves to choose what mode of consultation was best for them. This will require detailed triaging to ensure the consultation modality meets the clinical requirements necessary from the interaction. Recent work undertaken by Greenhalgh et al., involves virtual consultations that have been recorded, reflected upon and analysed for further training to develop the skills required when interacting using VC modalities [10].

### Study limitations

One of the main limitations to this research study is recruitment bias. As the question was related to the experience of HCP of VC within oncology and palliative care, and the survey was distributed on social media, one can assume that the participants were likely well attuned to information technology and relatively confident in their IT skills.

Within the limitations it is important to be aware of the researcher’s role within the research itself which may introduce an element of bias. As the researcher was a palliative medicine trainee with experience in VC, one could argue that this may have added an element of bias to the questions asked. On the other hand, the questions were based on current literature review.

### Recommendations

The findings from this study supplement the existing research with regard to the role of telemedicine and VC in delivering health care, especially since the Covid-19 pandemic. It draws attention to the limitations of VC within these fields and the requirement of further exploration of patient’s experience on certain aspects of the consultation. As part of ongoing work we hope to evaluate patient and carers views on VC via a large survey.

The findings have been shared in workforce planning meetings in our local Health boards and NHS Trusts, to consider how the implementation of VC within some aspects of clinical consultations can impact on consultation efficiency, workforce, and cost. Organisational meetings now have virtual meetings and consultations as standing agenda items, invariably containing a mixed bag of positive, indifferent, and negative feedback about various aspects of this new practice. The findings from this research can further evidence discussions and impact on care delivery of oncological and palliative care patients in future.

### Electronic supplementary material

Below is the link to the electronic supplementary material.

**Supplementary Material 1**: 1. Inclusion and exclusion criteria. 2. The survey. 3. Table of results (demographic and quantitative data). 4. Table summary of types of assessments undertaken by varying HCP’s using VC.

## Data Availability

All data generated or analysed during this study are included in this published article.

## Abbreviations

VC: (Virtual consultation)
HCP: (healthcare professional)
