# Supplementary material for: Virtual consultations: the experience of oncology and palliative care healthcare professionals
Source: BMC Palliat Care. 2024 May 2;23:114. doi: 10.1186/s12904-024-01400-y (PMC11064317; doi:10.1186/s12904-024-01400-y)
Supplement: Supplementary file 1 — Supplementary Material 1: 1. Inclusion and exclusion criteria. 2. The survey. 3. Table of results (demographic and quantitative data). 4. Table summary of types of assessments undertaken by varying HCP’s using VC. [file 12904_2024_1400_MOESM1_ESM.docx]

**Appendix**

Appendix 1: Inclusion and exclusion criteria

| Inclusion criteria | Exclusion criteria |
| --- | --- |
| - Participant is aged 18 or over | - Healthcare professional working outside of the fields of oncology or palliative medicine |
| - Participant is a healthcare professional in the speciality of oncology or palliative medicine anywhere in the world | - Participant that does not want to give consent or take part in the survey |
| - Participant has undertaken virtual consultations in the last 18 months | - No experience of undertaking virtual consultations |

Appendix 2: Survey


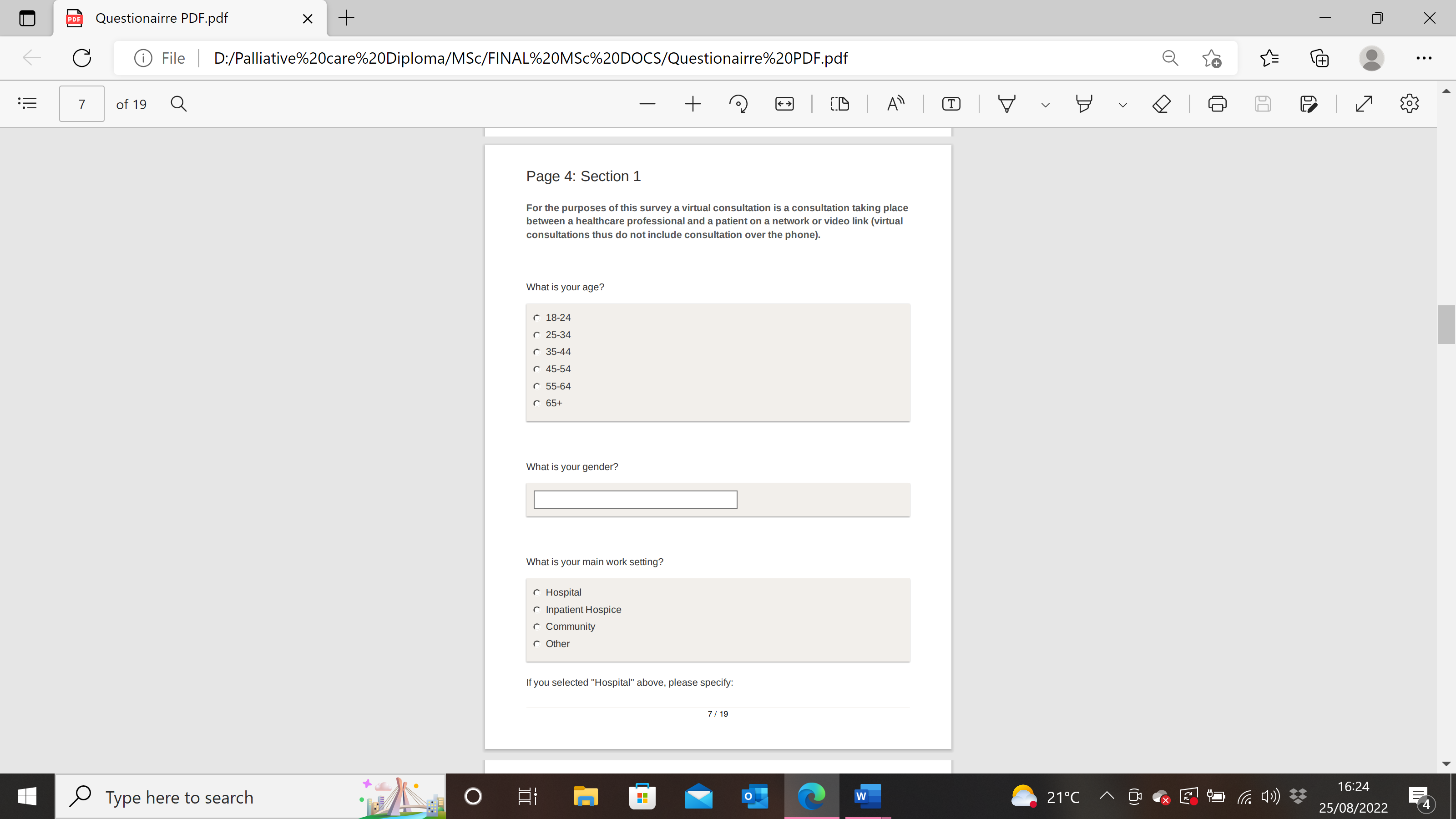


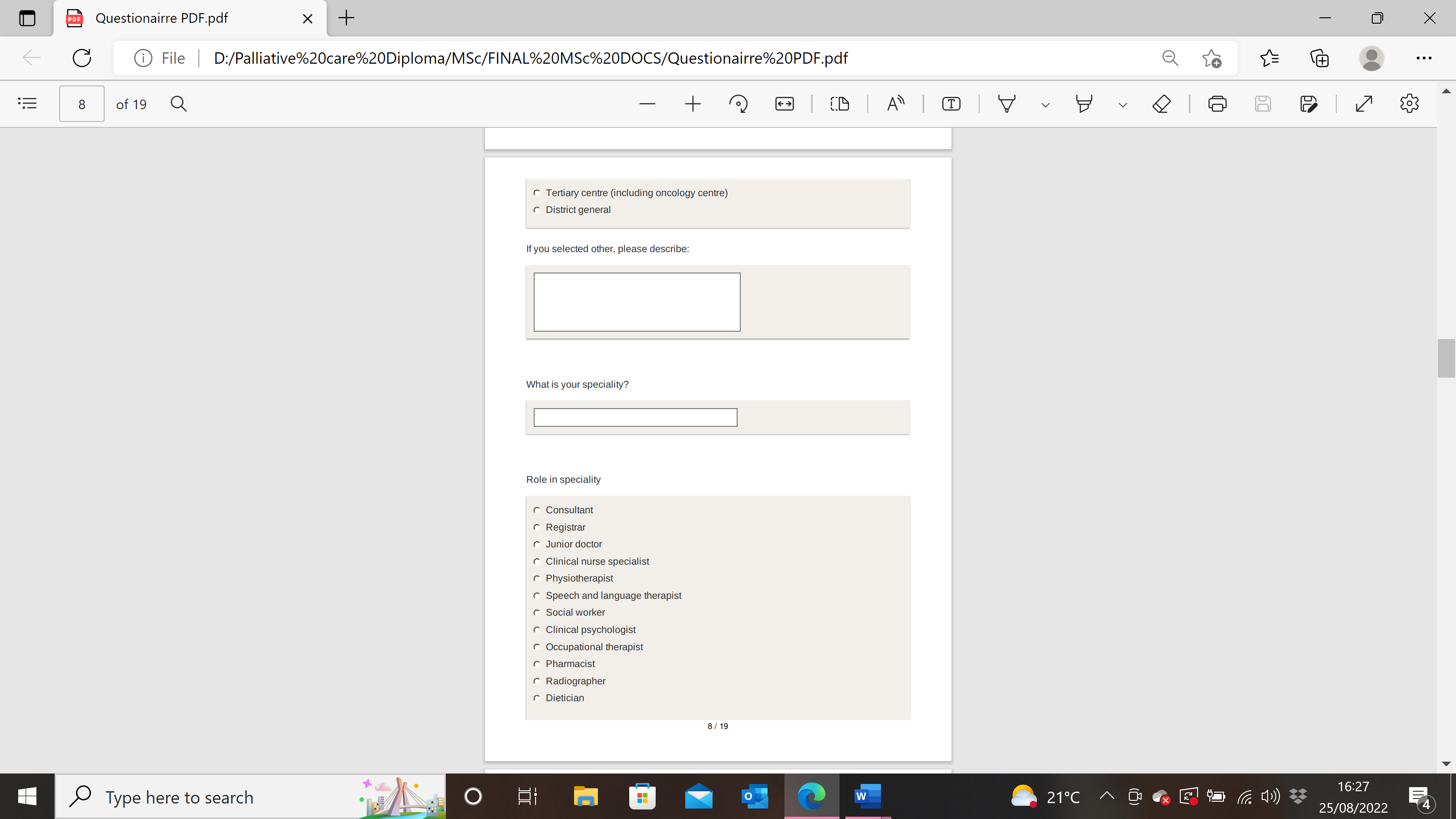


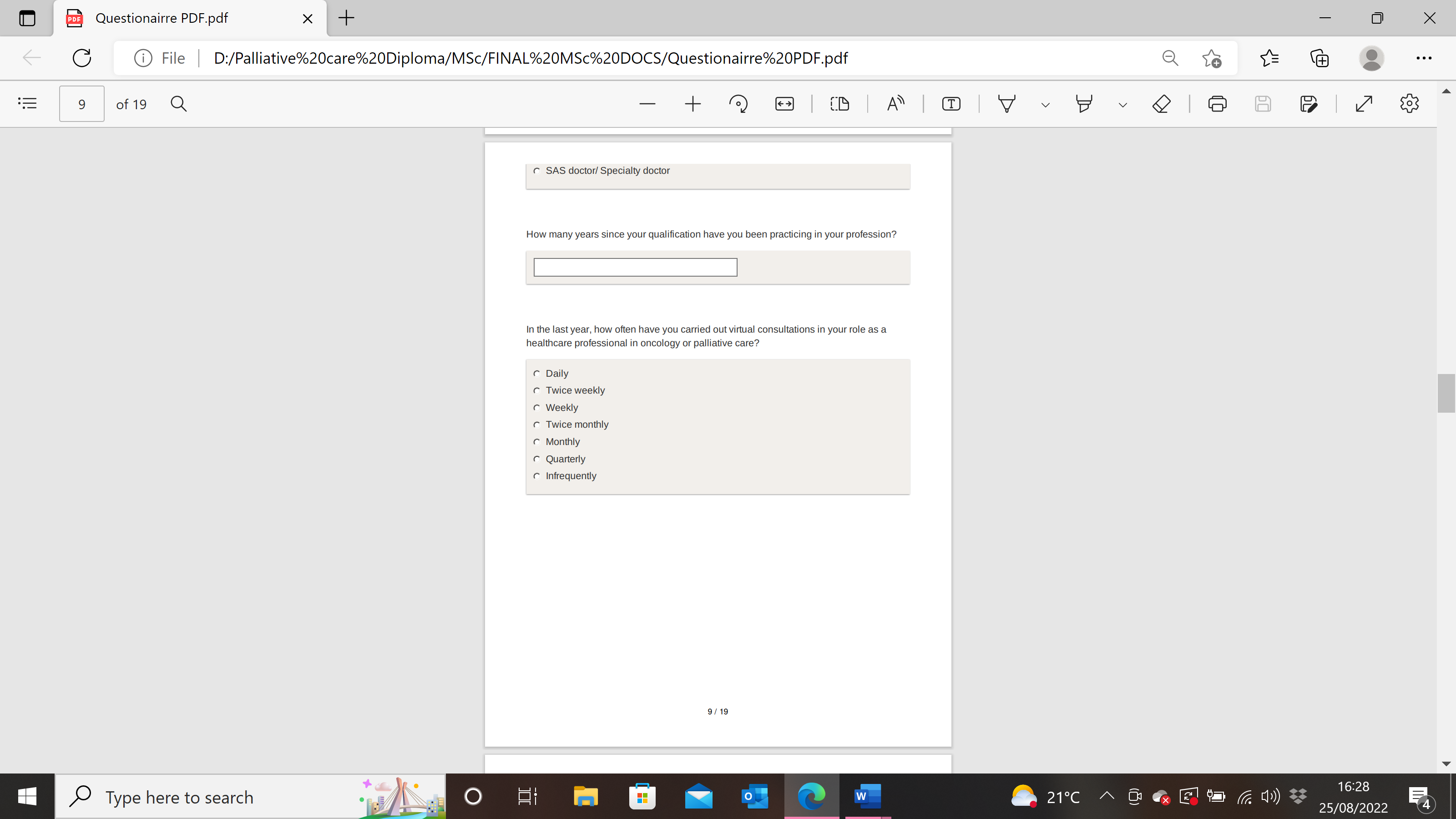


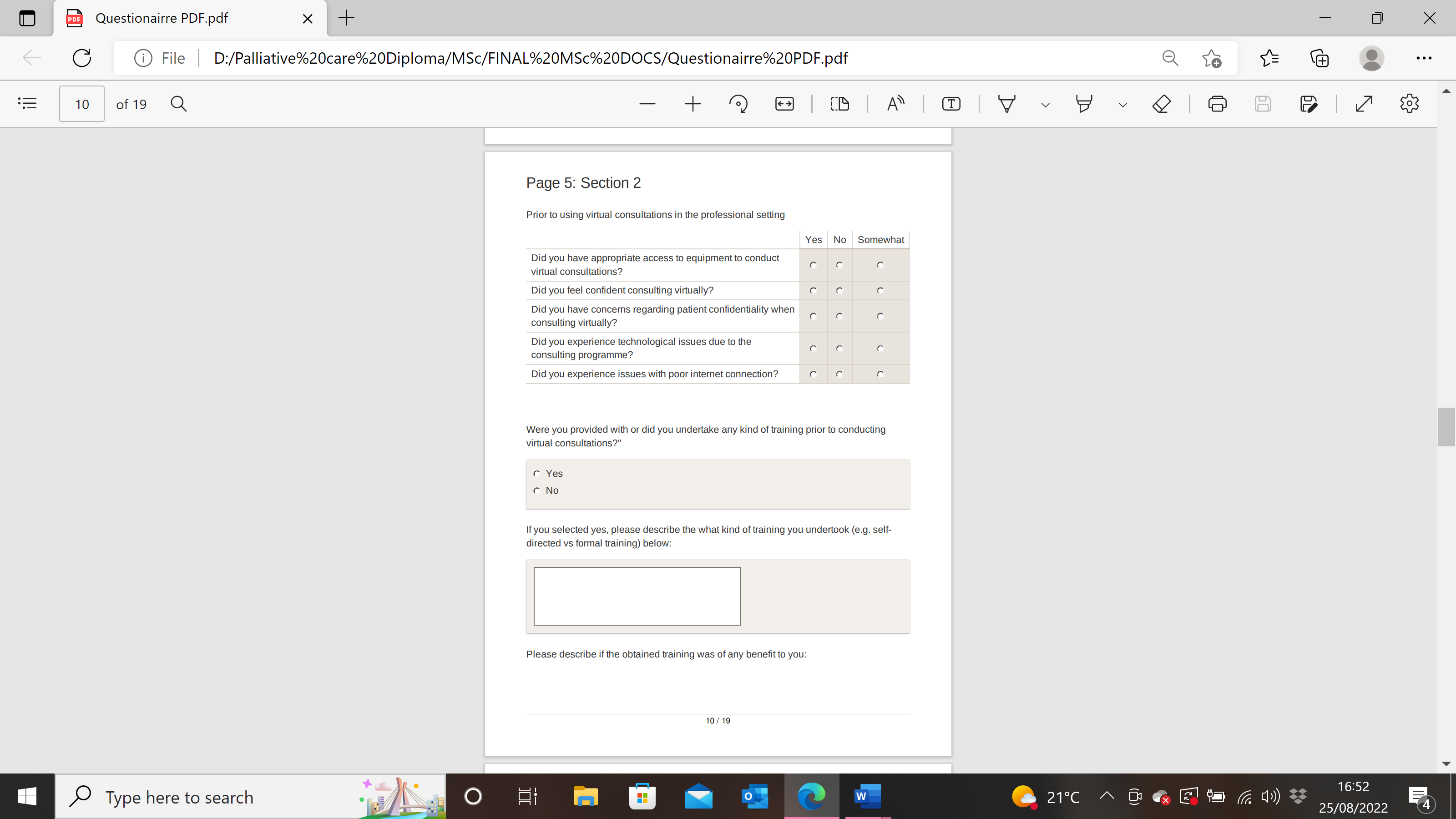


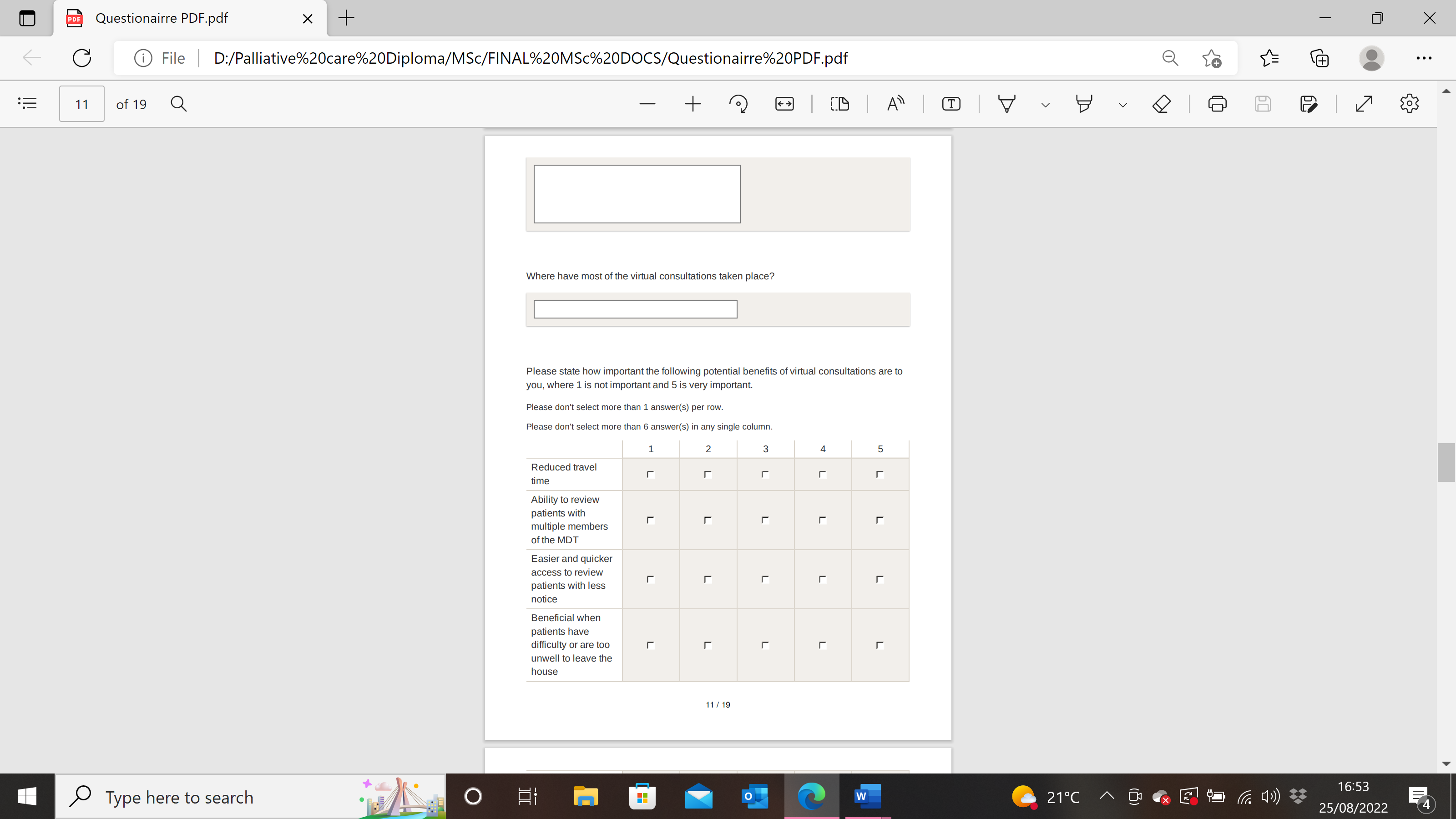


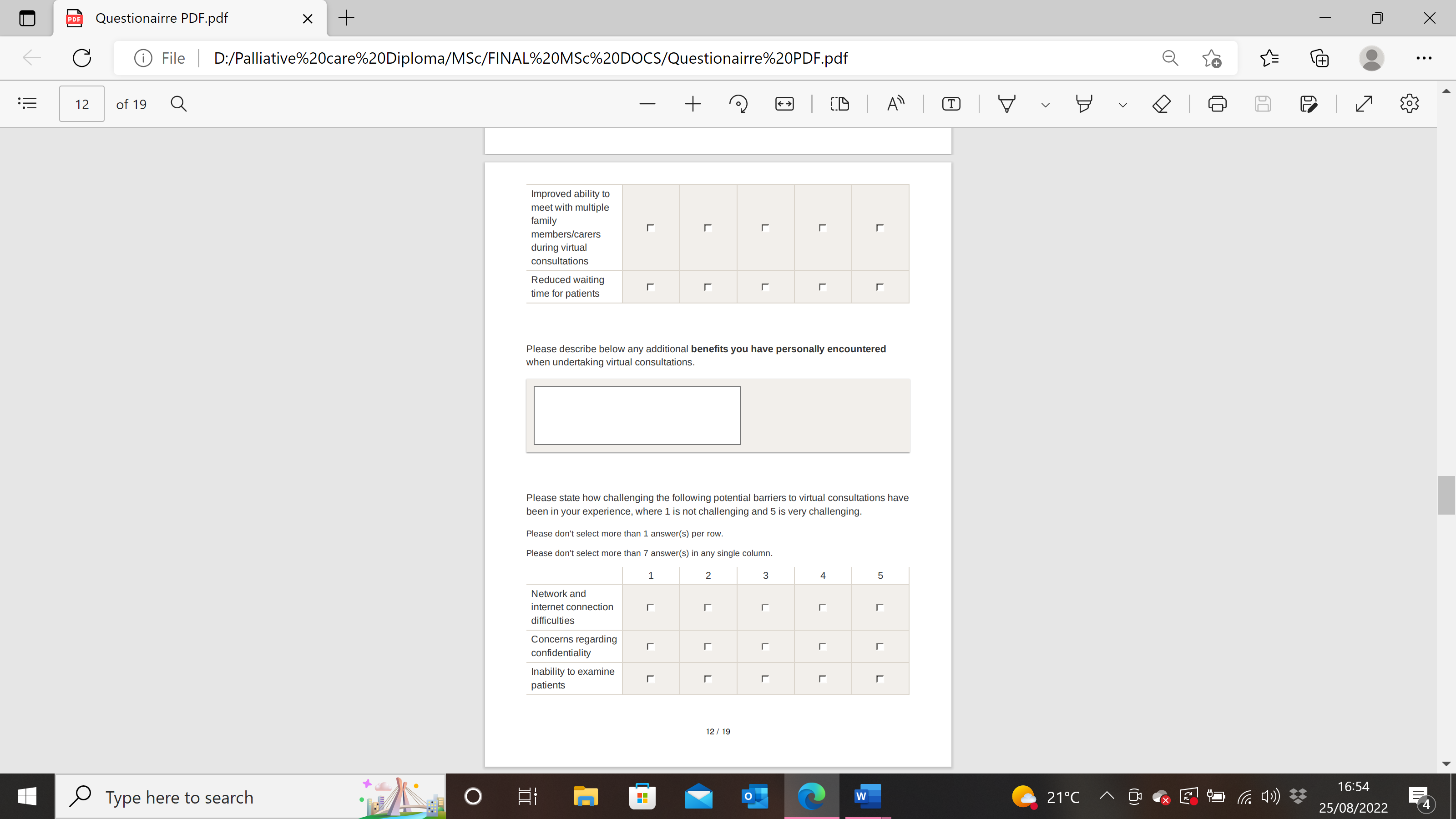


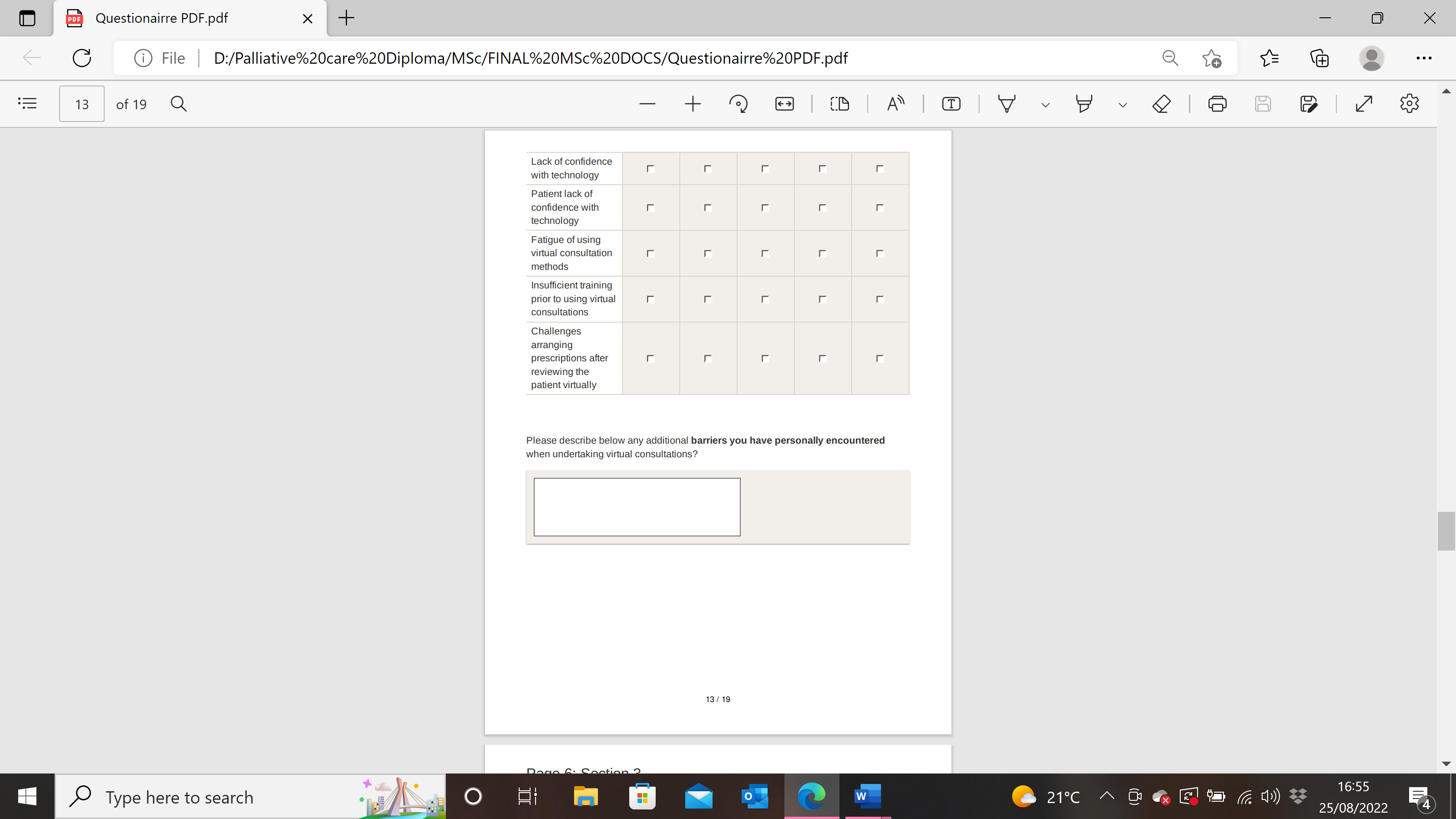


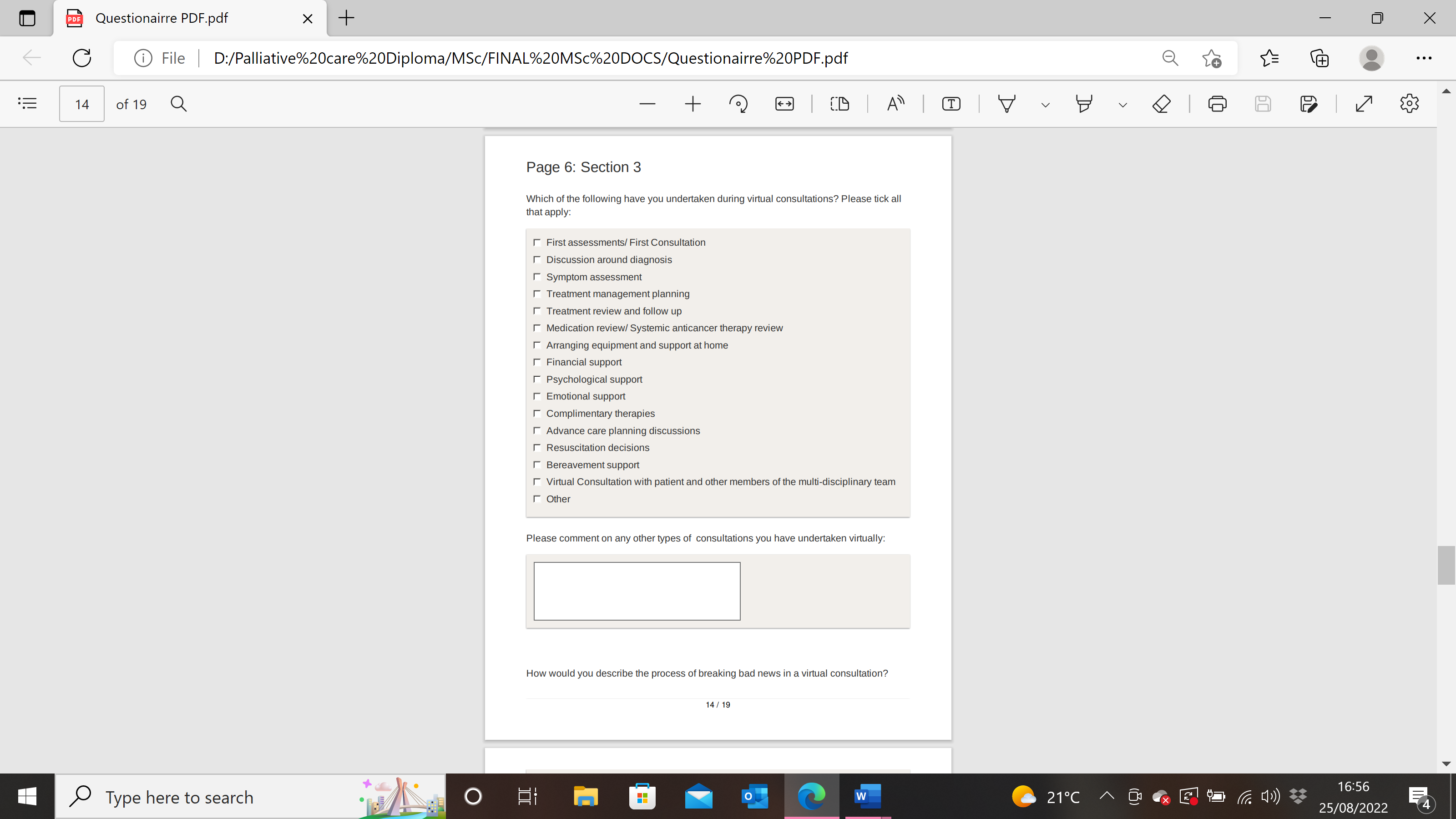


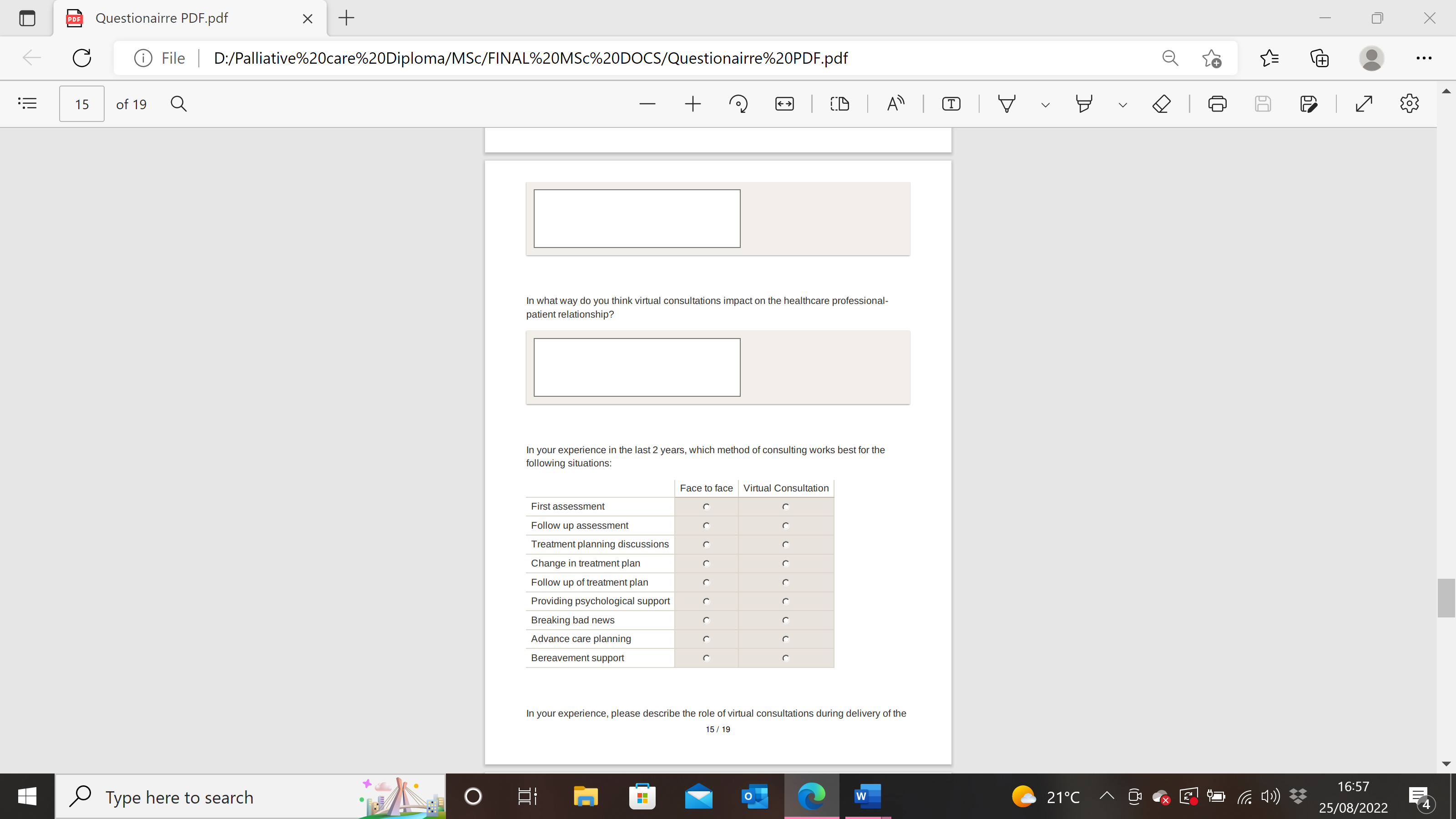


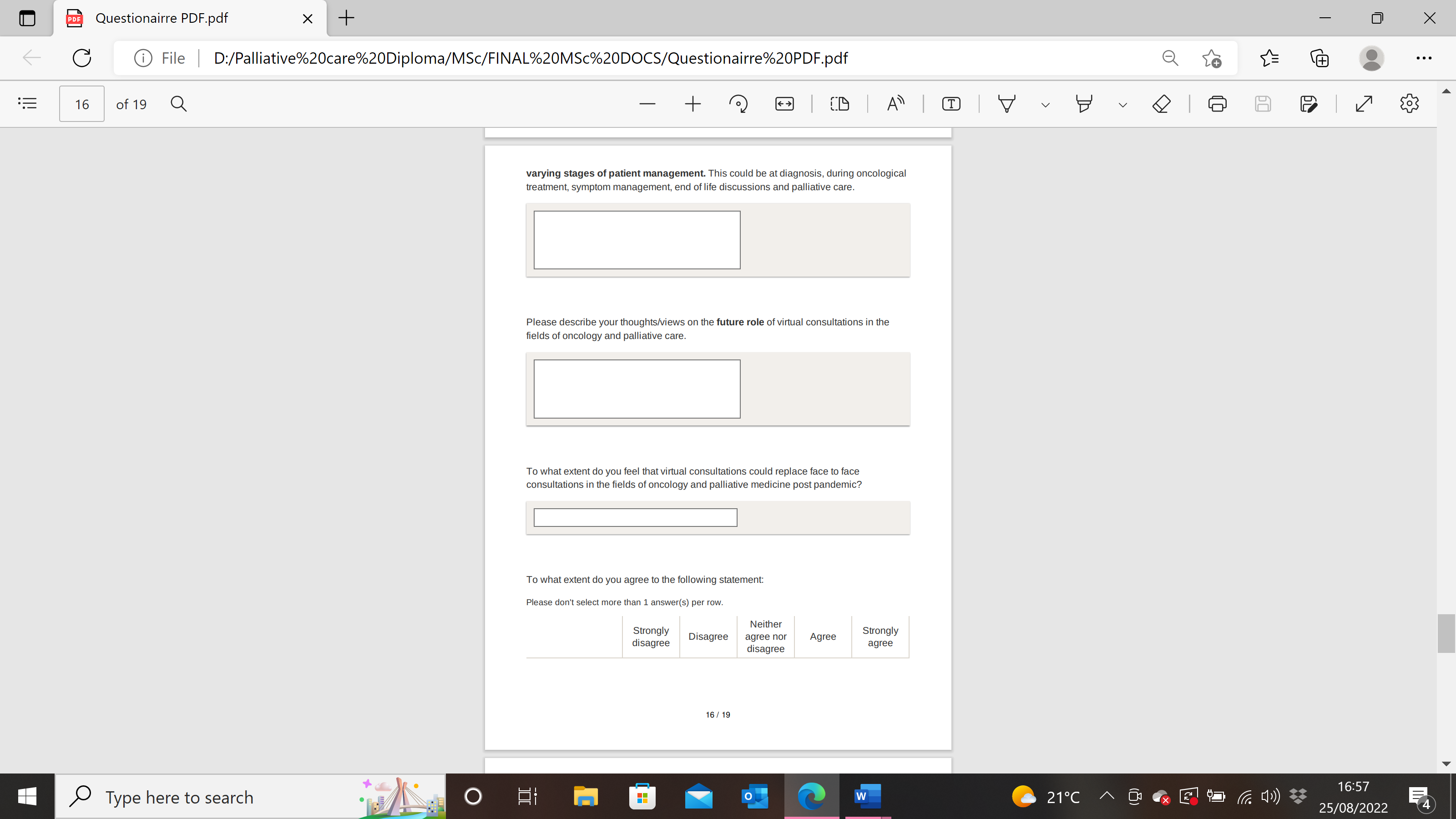


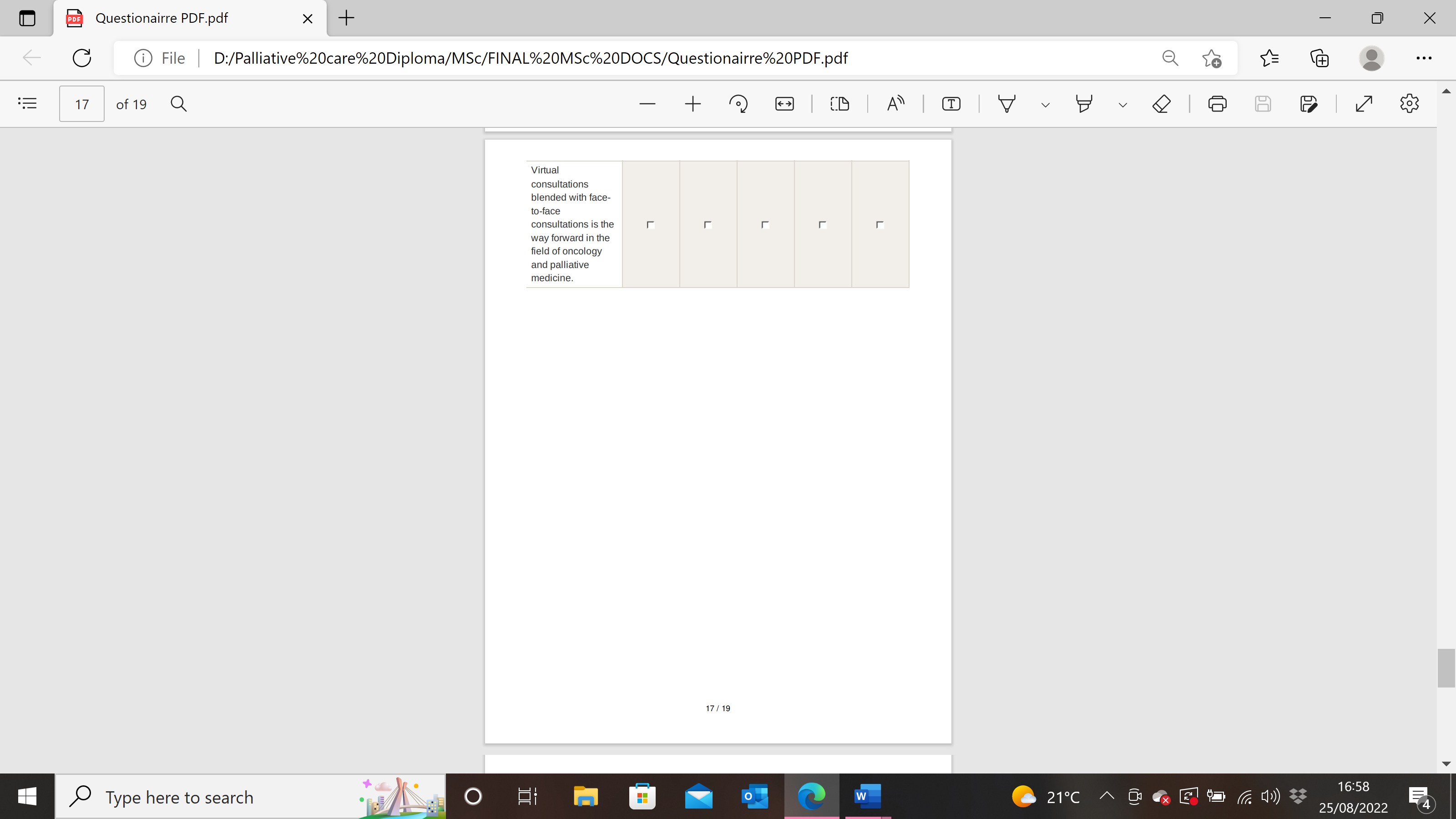


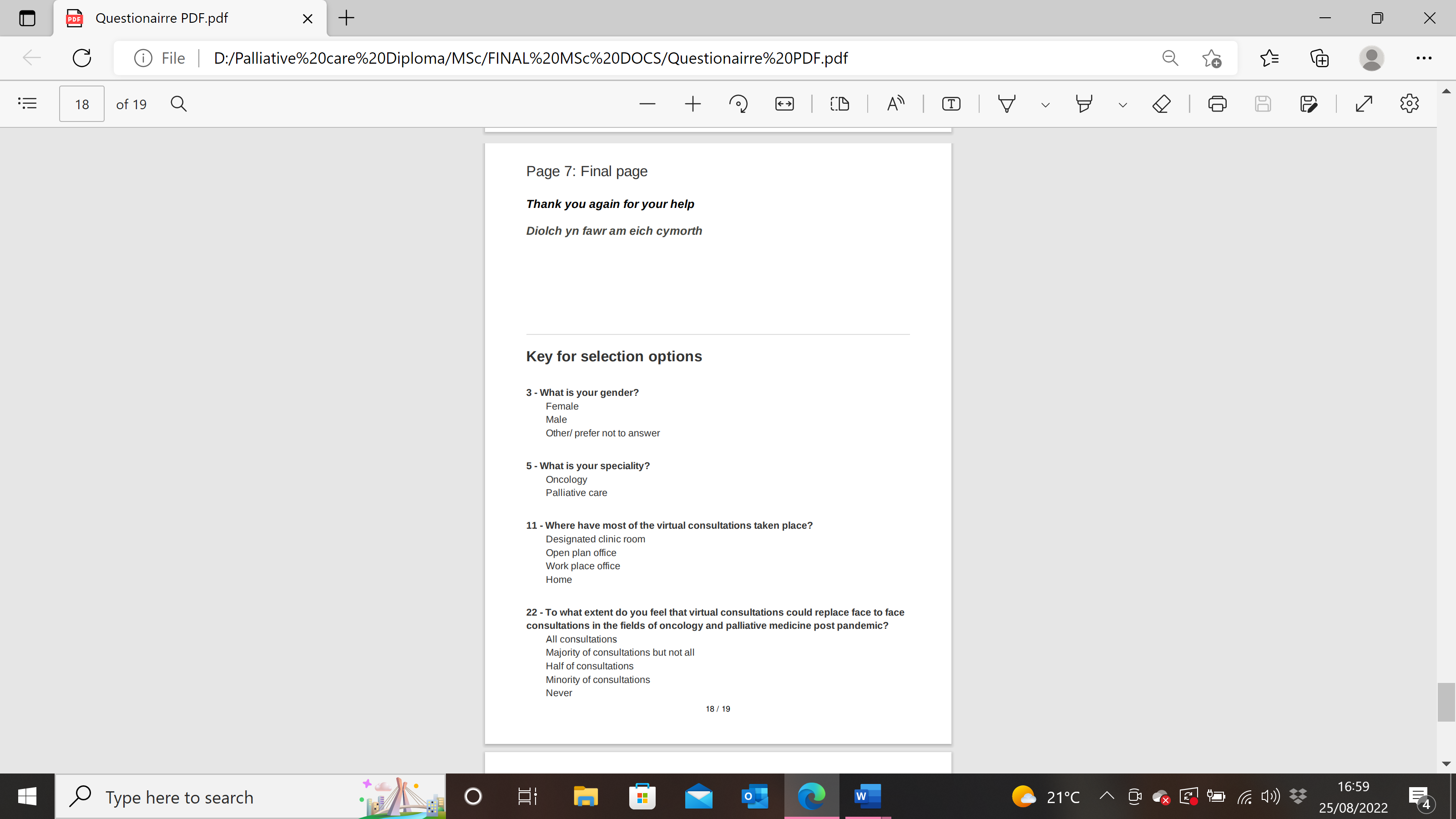


Appendix 3: Table of results

|  | **Number (%)** | **Percentage (%)** |
| --- | --- | --- |
| **Age (n=87)** |  |  |
| 18-24 | 2 | 2.3% |
|  |  |  |
| 25-34 | 9 | 10.3% |
| 35-44 | 30 | 34.5% |
| 45-54 | 35 | 40.2% |
| 55-64 | 10 | 11.5% |
| 65+ | 1 | 1.1% |
| **Gender** |  |  |
| Female | 66 | 75.9% |
| Male | 21 | 24.1% |
| **Setting** |  |  |
| Hospital | 55 | 63.2% |
| Inpatient hospice | 7 | 8% |
| Community | 24 | 27.6% |
| Other | 1 | 1.1% (equally divided between hospice, hospital and community palliative care) |
| **Of those in a Hospital setting (n=55)** |  |  |
| Tertiary centre (oncology centre) | 47 | 85.5% |
| District general | 8 | 14.5% |
| **Specialty (n=86)** |  |  |
| Oncology | 42 | 48.8% |
| Palliative care | 44 | 51.2% |
| **Role (n=86)** |  |  |
| Consultant | 37 | 43% |
| Registrar | 9 | 10.5% |
| Junior doctor | 0 | 0% |
| Clinical nurse specialist | 19 | 22.1% |
| Physiotherapist | 0 | 0% |
| Speech and language therapist | 5 | 5.8% |
| Social worker | 1 | 1.2% |
| Clinical psychologist | 1 | 1.2% |
| Occupational therapist | 2 | 2.3% |
| Pharmacist | 4 | 4.7% |
| Radiographer | 3 | 3.5% |
| Dietician | 0 | 0% |
| SAS/Specialty doctor | 5 | 5.8% |
| **Years since qualification (n=85)** |  |  |
| Mean |  | 17.89 years |
| Median |  | 18 years |
| **In the last year how often have you carried out VC in your role as a HCP (n=87)** |  |  |
| Daily | 6 | 6.9% |
| Twice weekly | 22 | 22% |
| Weekly | 21 | 24.1% |
| Twice monthly | 10 | 11.5% |
| Monthly | 4 | 4.6% |
| Quarterly | 4 | 4.6% |
| Infrequently | 20 | 23% |
| **PRIOR TO USING VC in the professional setting** |  |  |
| **Did you have appropriate access to equipment (n=87)** |  |  |
| Yes | 52 | 59.8% |
| No | 10 | 11.5% |
| Somewhat | 25 | 28.7% |
| **Did you feel confident consulting virtually (n=87)** |  |  |
| Yes | 49 | 56.3% |
| No | 16 | 18.4% |
| Somewhat | 22 | 25.3% |
| **Did you have concerns re patient confidentiality (n=87)** |  |  |
| Yes | 10 | 11.5% |
| No | 63 | 72.4% |
| Somewhat | 14 | 16.1% |
| **Did you experience technological issues due to the consulting programme (n=86)** |  |  |
| Yes | 39 | 45.3% |
| No | 25 | 28.7% |
| Somewhat | 22 | 25.6% |
| **Did you experience issues with poor internet connection (n=87)** |  |  |
| Yes | 39 | 44.8% |
| No | 31 | 35.6% |
| Somewhat | 17 | 19.5% |
| **Were you provided with training prior to conducting VC (n=87)** |  |  |
| Yes | 29 | 33.3% |
| No | 58 | 66.7% |
| **If yes to above: What kind of training (n=29)** | 29 | TEXT |
| **Was the training of any benefit to you (n=27)** | 27 | TEXT |
| **Where have most of the VC taken place (n=87)** |  |  |
| Designated clinic room | 39 | 44.8% |
| Open plan office | 9 | 10.3% |
| Work place office | 28 | 32.2% |
| Home | 11 | 12.6% |
| ***How important are these benefits of VC*** |  |  |
| **Reduced travel time (n=87)** |  |  |
| Not important | 16 | 18.4% |
| Slightly unimportant | 8 | 9.2% |
| Indifferent | 15 | 17.2% |
| Slightly important | 19 | 21.8% |
| Very important | 29 | 33.3% |
| **Ability to review patients with multiple members of MDT (n=87)** |  |  |
| Not important | 20 | 23% |
| Slightly unimportant | 13 | 14.9% |
| Indifferent | 18 | 20.7% |
| Slightly important | 20 | 23% |
| Very important | 16 | 18.4% |
| **Easier and quicker access to review patients with less notice (n=86)** |  |  |
| Not important | 10 | 11.6% |
| Slightly unimportant | 9 | 10.5% |
| Indifferent | 22 | 25.6% |
| Slightly important | 25 | 29.1% |
| Very important | 20 | 23.3% |
| **Beneficial when patients have difficulty or are too unwell to leave the house (n=86)** |  |  |
| Not important | 13 | 15.1% |
| Slightly unimportant | 8 | 9.3% |
| Indifferent | 12 | 14% |
| Slightly important | 16 | 18.6% |
| Very important | 37 | 43% |

| **Improved ability to meet with multiple family members/carers during virtual consultations (n=86)** |  | |  |
| --- | --- | --- | --- |
| Not important | 8 | | 9.3% |
| Slightly unimportant | 14 | | 16.3% |
| Indifferent | 16 | | 18.6% |
| Slightly important | 27 | | 31.4% |
| Very important | 21 | | 24.4% |
| **Reduced waiting time for patients (n=86)** |  | |  |
| Not important | 19 | | 21.8% |
| Slightly unimportant | 8 | | 9.2% |
| Indifferent | 13 | | 14.9% |
| Slightly important | 21 | | 24.1% |
| Very important | 26 | | 29.9% |
| **Additional *benefits encountered* undertaking VC** | 61 | | TEXT |
| ***How challenging are these potential barriers to VC*** |  | |  |
| **Network and connection difficulties (n=87)** |  | |  |
| Not challenging | 8 | | 9.2% |
| Slightly challenging | 17 | | 19.5% |
| Indifferent | 28 | | 32.3% |
| More challenging than not | 22 | | 25.3 |
| Very challenging | 12 | | 13.8 |
| **Concerns re confidentiality (n=87)** |  | |  |
| Not challenging | 37 | | 42.5% |
| Slightly challenging | 26 | | 29.9% |
| Indifferent | 18 | | 20.7% |
| More challenging than not | 5 | | 5.7% |
| Very challenging | 1 | | 1.1% |
| **Inability to examine patients (n=86)** |  | |  |
| Not challenging | 5 | | 5.8% |
| Slightly challenging | 9 | | 10.5% |
| Indifferent | 16 | | 18.6% |
| More challenging than not | 20 | | 23.3% |
| Very challenging | 36 | | 41.9% |
| **HCP Lack of confidence with technology (n=87)** |  | |  |
| Not challenging | 33 | | 37.9% |
| Slightly challenging | 24 | | 27.6% |
| Indifferent | 21 | | 24.1% |
| More challenging than not | 8 | | 9.2% |
| Very challenging | 1 | | 1.1% |
| **Patient lack of confidence of technology (n=86)** |  | |  |
| Not challenging | 4 | | 4.7% |
| Slightly challenging | 9 | | 10.5% |
| Indifferent | 25 | | 29.1% |
| More challenging than not | 35 | | 40.7% |
| Very challenging | 13 | | 15.1% |
| **Fatigue of using VC methods (n=87)** |  | |  |
| Not challenging | 20 | | 23% |
| Slightly challenging | 21 | | 24.1% |
| Indifferent | 22 | | 25.3% |
| More challenging than not | 17 | | 19.5% |
| Very challenging | 7 | | 8% |
| **Insufficient training prior to using VC (n=87)** |  | |  |
| Not challenging | 29 | | 33.3% |
| Slightly challenging | 26 | | 29.9% |
| Indifferent | 14 | | 16.1% |
| More challenging than not | 14 | | 16.1% |
| Very challenging | 4 | | 4.6% |
| **Challenges arranging prescriptions VC (n=84): *n=74 Prescribers*** |  | |  |
| Not challenging | 25 | | 29.8% |
| Slightly challenging | 17 | | 20.2% |
| Indifferent | 19 | | 22.6% |
| More challenging than not | 12 | | 14.3% |
| Very challenging | 11 | | 13.1% |
| **Barriers encountered undertaking VC** | 49 | | TEXT |
| **Which of the following have you undertaken during VC** |  | |  |
| First assessment | 56 | | 64.4% |
| Discussion around diagnosis | 45 | | 51.7% |
| Symptom assessment | 68 | | 78.2% |
| Treatment management planning | 55 | | 63.2% |
| Treatment review and follow up | 64 | | 73.6% |
| Medication review/SACT review | 52 | | 59.8% |
| Arranging equipment and support at home | 19 | | 21.8% |
| Financial support | 8 | | 9.2% |
| Psychological support | 33 | | 37.9% |
| Emotional support | 41 | | 47.1% |
| Complementary therapies | 2 | | 2.3% |
| ACP | 32 | | 36.8% |
| Resuscitation decisions | 18 | | 20.7% |
| Bereavement support | 7 | | 7% |
| VC with patient and MDT | 37 | | 42.5% |
| Other | 4 | | 4% |
| **Please comment on any other types of consultations you have undertaken virtually** | 9 | | TEXT |
| **How would you describe the process of breaking bad news in VC** | 70 | | TEXT |
|  | 57 had experience (N/A to role in 13 respondents) | |  |
| *Negative experience* | 37 | | 64.9% |
| *Positive experience* | 7 | | 12.2% |
| *Mixed experience* | 13 | | 22.8% |
| **In what way to you think VC impact on HCP patient relationship** | 71 | | TEXT |
| **In your experience in the last 2 years which method of consulting works best for the following situations** | F2F | VC |  |
| First Assessment | 79 | 5 | F2f 94% VC 6% |
| Follow up assessment | 30 | 53 | F2f 36.1% VC 63.9% |
| Treatment planning discussions | 54 | 23 | F2f 70.1% VC 29.9% |
| Change in treatment plan | 58 | 20 | F2f 74.4% VC 25.6% |
| Follow up treatment plan | 18 | 62 | F2f 22.5% VC 77.5% |
| Providing psychological support | 63 | 17 | F2f 78.8% VC 21.3% |
| Breaking bad news | 79 | 0 | F2f 100% VC 0% |
| ACP | 64 | 11 | F2f 85.3% VC14.7% |
| Bereavement support | 53 | 19 | F2f 73.6% VC 26.4 |
| **Please describe the role of VC during the varying stages of patient management** | 63 | | TEXT |
| **Please describe your thoughts views on the future role of VC in the fields of palliative care and oncology** | 71 | | TEXT |
| **To what extent do you feel that VC could replace f2f consultations in the field of oncology and palliative medicine post pandemic (n=86)** |  | | TEXT |
| All | 0 | | 0% |
| Majority of consultations but not all | 2 | | 2.3% |
| Half of consultations | 46 | | 53.5% |
| Minority of consultations | 35 | | 40.7% |
| Never | 3.5 | | 3.5% |
| **To what extent do you agree to the following statement (n=87)** |  | |  |
| Strongly disagree | 7 | | 8% |
| Disagree | 9 | | 10.3% |
| Neither agree or disagree | 9 | | 10.3% |
| Agree | 35 | | 40.2% |
| Strongly agree | 27 | | 31% |
|  |  | |  |

Appendix 4: Table summary of types of assessments undertaken by varying HCP’s using VC

| **Which of the following have you undertaken during VC** | Consultant | Registrar | CNS | Speech and Language therapist | Social worker | Clinical psychologist | OT | Pharmacist | Radiographer | SAS Dr/Specialty Dr |
| --- | --- | --- | --- | --- | --- | --- | --- | --- | --- | --- |
| First assessment | 27 | 5 | 9 | 4 | 1 | 1 | 2 | 0 | 1 | 5 |
| Discussion around diagnosis | 26 | 5 | 6 | 1 | 1 | 0 | 0 | 2 | 0 | 3 |
| Symptom assessment | 33 | 8 | 15 | 4 | 0 | 0 | 0 | 2 | 2 | 3 |
| Treatment management planning | 30 | 7 | 8 | 2 | 0 | 0 | 0 | 2 | 2 | 3 |
| Treatment review and follow up | 32 | 8 | 8 | 5 | 0 | 0 | 0 | 4 | 2 | 4 |
| Medication review/SACT review | 27 | 7 | 9 | 0 | 0 | 0 | 0 | 4 | 2 | 2 |
| Arranging equipment and support at home | 2 | 1 | 10 | 2 | 0 | 0 | 1 | 0 | 1 | 2 |
| Financial support | 1 | 0 | 6 | 0 | 1 | 0 | 0 | 0 | 0 | 0 |
| Psychological support | 8 | 1 | 13 | 2 | 1 | 1 | 2 | 1 | 2 | 1 |
| Emotional support | 11 | 1 | 16 | 3 | 1 | 1 | 2 | 1 | 2 | 2 |
| Complementary therapies | 0 | 0 | 0 | 1 | 0 | 0 | 0 | 0 | 1 | 0 |
| ACP | 14 | 2 | 10 | 1 | 1 | 0 | 0 | 0 | 0 | 3 |
| Resuscitation decisions | 9 | 2 | 5 | 0 | 0 | 0 | 0 | 0 | 0 | 1 |
| Bereavement support | 1 | 0 | 3 | 1 | 1 | 1 | 0 | 0 | 0 | 1 |
| VC with patient and MDT | 15 | 3 | 7 | 5 | 0 | 0 | 1 | 2 | 0 | 3 |
| Other | 1 | 0 | 2 | 1 | 0 | 0 | 0 | 0 | 0 | 0 |
| Total response | 237 | 50 | 127 | 31 | 7 | 4 | 8 | 18 | 15 | 33 |

*Further assessments included by participants included: swallow assessments and therapy reviews, administrative support for scheduling investigations or radiological tests, discharge planning and family meetings, review of patients at the end of life.
